# 

**Published:** 1882-12

**Authors:** 


ESTABLISHED IN 1855.

OLD SERIES.

Vol. XX—No. 9.

DECEMBER, 1882.

NEW SERIES.

Vol. II—No. 3.

S.G.O.
INDEXED

### ATLANTA

### MEDICAL REGISTER.

(*ATLANTA MEDICAL AND SURGICAL JOURNAL.*)

EDITED BY

JAMES B. BAIRD, M.D.

H. H. DICKSON, PUBLISHER.   TERMS, $2.50 A YEAR IN ADVANCE.

ATLANTA, GEORGIA:
*H. H. DICKSON, BOOK AND JOB PRINTER AND BOOKBINDER.*
1882.

